# Pilot study: evaluation of the use of the convergent interview technique in understanding the perception of surgical design and simulation

**DOI:** 10.1186/1916-0216-42-40

**Published:** 2013-06-19

**Authors:** Heather Logan, Johan Wolfaardt, Pierre Boulanger, Bill Hodgetts, Hadi Seikaly

**Affiliations:** 1 Institute for Reconstructive Sciences in Medicine, Misericordia Community Hospital Edmonton, 1W-02, 16940-87 Avenue, Edmonton, AB T5R 4H5, Canada; 2 Department of Computing Science, University of Alberta, Athabasca Hall, Room 411, Edmonton, AB T6G 2E8, Canada; 3 Speech Pathology and Audiology, 2–16, Corbett Hall Edmonton, University of Alberta, 116 St. and 85 Ave, Edmonton, AB T6G 2G4, Canada; 4 Department of Otolaryngology, Head & Neck Surgery, 8440 - 112th Street, 1E4.34 WMC, Edmonton, AB T6G 2B7, Canada

**Keywords:** Convergent interview technique, Surgical design and simulation, Medical models, Head and neck reconstruction

## Abstract

**Background:**

It is important to understand the perceived value of surgical design and simulation (SDS) amongst surgeons, as this will influence its implementation in clinical settings. The purpose of the present study was to examine the application of the convergent interview technique in the field of surgical design and simulation and evaluate whether the technique would uncover new perceptions of virtual surgical planning (VSP) and medical models not discovered by other qualitative case-based techniques.

**Methods:**

Five surgeons were asked to participate in the study. Each participant was interviewed following the convergent interview technique. After each interview, the interviewer interpreted the information by seeking agreements and disagreements among the interviewees in order to understand the key concepts in the field of SDS.

**Results:**

Fifteen important issues were extracted from the convergent interviews.

**Conclusion:**

In general, the convergent interview was an effective technique in collecting information about the perception of clinicians. The study identified three areas where the technique could be improved upon for future studies in the SDS field.

## Background

It is important to understand the perceived value of surgical design and simulation (SDS) amongst surgeons, as this will influence its implementation in clinical settings. In discussing the utility of rapid prototyped medical models (a highly accurate physical three-dimensional model representing the anatomy of a human derived from the rapid prototyping printing technique), many researchers have evaluated perceived usefulness through the development of questionnaires directed toward surgeons and other interest groups and through case studies [1,2]. Although these studies have helped to better understand the benefits that rapid prototyped medical models bring to different subject groups that utilize them, no researchers have yet examined the utility of virtual surgical planning (VSP) technology and medical models using the convergent interview technique.

In the present study, the convergent interview technique was used to gather information and examine the perceptions of clinicians in head and neck reconstruction regarding the utility of VSP and medical models in their practice. The primary objective of the present study was to explore the application of the convergent interview research method in the field of SDS. The secondary objective of the convergent interview was to evaluate the perceptions of head and neck surgeons in regards to medical models and VSP technologies in their practice of mandibular reconstruction and to understand whether the convergent interview technique would bring out new perceptions not discovered by other qualitative collection techniques.

## Methods

Five surgeons experienced in microvascular reconstruction were asked to participate in the study by means of purposive sampling. Purposive sampling was used due to the limited number of head and neck surgeons in Edmonton, Alberta as well as the limitations of the scope of the project. The optimal sampling size was data-driven rather than predetermined [3]. The optimal sample size is determined when stability is reached, which occurs when agreement among all interviewees is achieved and disagreement between them is explained on all the issues raised [3].

**Figure 1:**
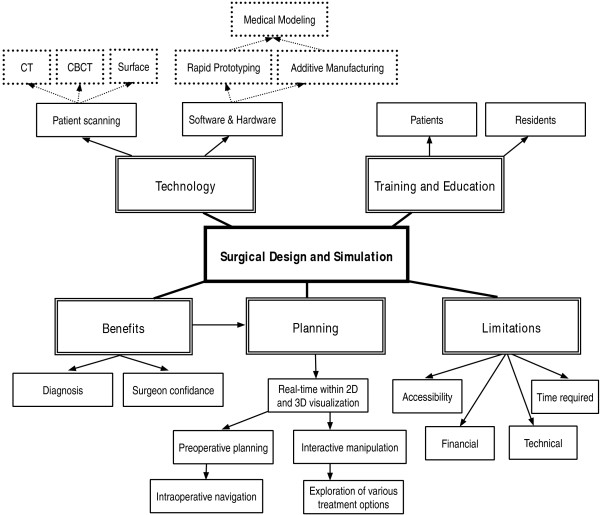
Mind map diagram of information to be used for convergent interview.

The interviewer gained prior knowledge of the research topic of interest by doing some initial reading, brainstorming and mind-mapping (Figure 1). By gaining prior knowledge, the researcher was able to develop an appropriate opening question; the researcher gained confidence before conducting the initial interview and was able to establish rapport and maintain interview dynamics with the respondents [3-5].

The following steps outline the planning and management issues of the convergent interviewing technique based mainly on the steps recommended by Dick (1990) and by Rao and Perry (2003). The first step was to establish initial contact with potential participants. After being given an overview of the research and the purpose of the interview, the respondents were asked to participate in the interview. After agreement, the venue and time were decided. All interviews were conducted face-to-face in the Medical Modeling Research Laboratory (MMRL) of the Institute for Reconstructive Sciences in Medicine (iRSM) at the Misericordia Community Hospital in Edmonton, Alberta, Canada.

Each interview began with the researcher introducing the project and clarifying issues of confidentiality before the interview. The interviewee was asked for permission to record the interview using a digital audio recorder. The researcher then asked an opening question which was framed to encourage the interviewee to reveal their attitudes about the topic of interest without implying constraints on the nature of the response. The opening question was also intended to encourage the respondent to discuss their own experiences in a way that is meaningful to them [6]. The opening question used was: “I’m interested in learning about the perceived utility of medical modeling and computer-assisted planning software. Tell me what you think of these two tools in your practice.” This opening question allowed the respondents to open up and talk about their opinions. Probe questions were used to help keep interviewees talking and the interview focused. Probe questions were developed before each interview but only after the first one, based on the preceding interview. A list of the probe questions were:

1. “Can you give me an example of this?”

2. “Can you elaborate a little?”

3. “What exactly did you mean by…?”

4. “Is that all? Is there anything you missed out?”

5. “How does that compare with what you said before?”

6. “What are the pros and cons of this situation?”

7. “And how did you feel about that?”

8. “Why do you think this is the case?”

9. “What would have to change in order for…?”

10. ” How was… different from…?”

11. “What sort of an impact do you think…?”

12. “What criteria did you use to…?”

13. “How did you decide/determine/conclude…?”

14. “What is the connection between…and…?”

15. “How might your assumptions about…have influenced how you are thinking about…?”

As the interview neared completion, the interviewer invited the respondent to review key points from what was discussed. The researcher asked questions such as, “of all the issues you have mentioned what are the most and least important issues?” and “Could you please prioritize them in order of importance?”. When the interviewee could no longer add further information, the interviewer summarized the interview to confirm the responses. The interviewer then reviewed what would happen to the information.

All interviews were recorded using a digital recording software program as suggested by Riege et al. [3]. Recording was done in order to increase the accuracy of the data collection process, permitting the interviewer to be more attentive to the interviewee. Recording the interviews allowed the interviewer to replay the recordings, which helped the researcher interpret the data, identify important themes and correct and expand on the interview notes [3].

Ethics approval was granted by the Health Research Ethics Board - Health Panel under reference number Pro00018582.

## Results

A total of five interviews were needed to reach stability between the interviews. Each interview lasted between 30 minutes to 60 minutes. A total of 3 hours of interview time was conducted. Tables 1, 2, 3 and 4 outline the important issues that were extracted for the convergent interviews.

**Table 1:** Important issues extracted from the interviews about the utility of medical modeling and computer-assisted planning software

	
1.	Useful tool in planning and exploring other options.
2.	Helps with teaching and learning for students and patients.
3.	Accuracy, efficiency, quality are all potential advantages.
4.	Potential for improved functional outcome: planning for dental implants, dental rehabilitation, better occlusion, less TMJ dysfunction and improved mandibular strength and cosmetic outcome.
5.	Potential for reducing operating room time.
6.	Communication between team members can be facilitated by the technology.
7.	Increase in surgeon confidence.
8.	Initial cost of the setup of the technology is a disadvantage but can be outweighed by the longterm benefits.
9.	Accessibility and availability of the resource is a disadvantage.
10.	Time constraint is a disadvantage. This includes time to learn the technology, collaborate and plan each case and turnaround and production time between initial case planning and scheduled surgery.
11.	There is minimal quantitative research available to prove the accuracy, benefits and functional outcome of the patients when digital surgical design and simulation is used. There is also a need for a cost benefit analysis.
12.	Surgical design and simulation can bring more of a team approach.
13.	Surgical design and simulation is not included in medical student training.
14.	There is cultural resistance to the surgical design and simulation technology.
15.	The field of surgical design and simulation is a work in progress.

**Table 2:** Agreements & disagreements on various issues raised by the respondents

**Participant**	**A**	**B**	**C**	**D**	**E**
**Issue**					
**1**	Y	Y	Y	Y	Y
**2**	O	O	Y	X	Y
**3**	Y	Y	Y	Y	Y
**4**	Y	Y	Y	Y	O
**5**	O	Y	Y	Y	Y
**6**	Y	Y	Y	Y	Y
**7**	Y	Y	Y	Y	Y
**8**	O	Y	Y	Y	X
**9**	Y	Y	Y	Y	Y
**10**	Y	Y	Y	Y	Y
**11**	O	Y	Y	Y	Y
**12**	Y	Y	Y	Y	Y
**13**	Y	Y	Y	Y	Y
**14**	Y	Y	Y	Y	Y
**15**	Y	Y	Y	Y	Y

Key:

Y = Agreements.

X = Disagreements.

0 = Not familiar with issue so neither agreement or disagreement.

**Table 3:** Overview of the respondent’s “Agreements”, “Disagreements”, and “not familiar issues or not mentioned”

	**Agreements (%)**	**Disagreements (%)**	**Not familiar issues or Not mentioned (%)**
**Issue**			
1	100	-	-
2	40	20	40
3	100	-	-
4	80	-	20
5	80	-	20
6	100	-	-
7	100	-	-
8	60	20	20
9	100	-	-
10	100	-	-
11	80	-	20
12	100	-	-
13	100	-	-
14	100	-	-
15	100	-	-

**Table 4:** Issues discussed in the interviews and issues raised in the literature

	**Issue**	**Reported by the interviewee**	**Reported in the literature**
**1**	Useful tool in planning and exploring other options.	✓	(8,9)
**2**	Helps with teaching and learning for students and patients.	✓	(8,10)
**3**	Accuracy, efficiency, quality are all potential advantages.	✓	(11,12,12-14)
**4**	Potential for improved functional outcome: planning for dental implants, dental rehabilitation, better occlusion, less TMJ dysfunction and improved mandibular strength and cosmetic outcome.	✓	(14,15)
**5**	Potential for reducing Operating Room time.	✓	(16,17)
**6**	Communication between team members can be facilitated by the technology.	✓	(9)
**7**	Increase in surgeon confidence.	✓	(17)
**8**	Initial cost of the setup of the technology is a disadvantage but can be outweighed by the longterm benefits.	✓	(18)(19)
**9**	Accessibility and availability of the resource is a disadvantage.	✓	(17)
**10**	Time constraint is a disadvantage which includes time to learn the technology, collaborate and plan each case and turnaround and production time between initial case planning and scheduled surgery.	✓	(18)
**11**	There is minimal quantitative research available to prove the accuracy, benefits and functional outcome of the patients when digital surgical design and simulation is used. There is also a need for a cost-benefit analysis.	✓	(20)
**12**	Surgical design and simulation can bring more of a team approach.	✓	(17)
**13**	Surgical design and simulation is not included in medical student training.	✓	
**14**	There is cultural resistance to the surgical design and simulation technology.	✓	
**15**	The field of surgical design and simulation is a work in progress.	✓	

The group of respondents who participated varied in their experience level, in their experiences of their personal practice and in their training, and these factors added variety in the responses and opinions observed.

Based on the interview results, issues 1, 3, 6, 7, 9, 10, 12, 13, 14 and 15 all had 100% agreement among the participants (Tables 2 and 3). Disagreement was found on issues 2 and 8.

### Disagreement on issue 2: helps with teaching and learning for students and patients

The participant disagreed with the idea of using medical models and virtual surgical planning to teach students. The participant said that, “Students need to learn more about the art of surgery. If they are trained with the technology, they may not be able to perform reconstruction without the tools.”

### Disagreement on issue 8: initial cost of the setup of the technology is a disadvantage but can be outweighed by the long-term benefits

The participant said: “What is the answer? We don’t know that. In theory you would hope that the operative time is less so there would be less cost [and] that the potential complications down the road would be less so there is a savings there versus the upfront costs of the models and the time required for the people to create them.”

With issues 4, 5, 8 and 11, one participant was either not familiar with the issue or did not mention the issue throughout the interview. Issue 2 had two such participants. Of the fifteen important issues extracted from interviews, eight were categorized as an advantage of the utility of medical modeling and computer-assisted planning software, while six were categorized as a disadvantage and one was categorized as neutral.

## Discussion

### Interpretation of the outcomes

The purpose of the present study was to examine the application of the convergent interview technique in the field of SDS and evaluate whether the technique would bring out new perceptions of virtual surgical planning and medical models not discovered by other qualitative collection techniques. The interviews followed the convergent interview protocol as planned by the researcher. The discussions remained focused and allowed the interviewees to talk about their personal opinions. The interviews also allowed the interviewees to talk about some personal experiences, which was an important feature of the technique, although it did not occur as much as anticipated.

The interviewer recognized that many of the initial points that were discussed by the interviewees were general observations on the technology that are often repeated in the literature. The interviewer often had to probe the interviewee with questions that pushed the interviewees to talk about their personal opinions and experiences rather than general issues that they had probably read of or heard about but had not experienced themselves.

One of the significant findings from each interview was that every surgeon reported that they had little or no experience using the surgical planning software, which is a significant part of the SDS process. Using the software or participating in the digital simulation of the planning gives the clinician an understanding of the anatomy of the patient in three dimensions (3D) and an opportunity to explore surgical options. Of the five interviewees, two had never seen or used the surgical planning software, two had only observed it being used and one had used it in a workshop setting. After close inspection of all of the interviews, after completion of the convergent interview process, the researcher observed that many of the statements made by the interviewees were likely not their personal opinions based on personal experience, but appeared to have been based on issues reported in the literature. Table 4 outlines the issues discussed in the interviews and links them to the issues raised in the literature. Of the fifteen issues raised, only three issues did not appear to be outlined in the literature. The issues were; (1) surgical design and simulation is not included in resident training; (2) there is cultural resistance to the surgical design and simulation technology and; (3) the field of surgical design and simulation is a work in progress.

As a consistent user of 3D software, it was difficult for the interviewer to understand how the interviewees could have responded as they did without having physically used and experienced the surgical planning process using the software. Although not all issues extracted for the interview are related to personal experience, many are associated. This finding would have been very useful in guiding the interviews after the first one, but was not realized until completion of the process.

A possible explanation for this trend in the interviews is related to the fact that all participants were from Edmonton, Alberta, Canada, where the technology is more accessible than many other locales and knowledge about the technology is more accessible because of the MMRL at iRSM. The participants are also all from the same field of work and are closely associated as clinicians. The surgeons attitudes and perceptions of the technology may have been influenced by each others’ associations; that is, through social learning. This interpretation of the researcher is based on the balance theory, which holds that humans organize their attitudes in a symmetrical way [7]. This theory is associated with the idea that humans turn to others to obtain consensual validation of their views [7].

## Conclusion

The first objective of the present study was to explore the application of the convergent interview research method in the field of VSP and medical models in mandibular reconstruction. In general, the convergent interview was an effective technique in collecting information about the perception of clinicians. There were three areas of the technique that need to be improved upon for future studies in this field. The first is an improved opening question to direct and guide the participants to discuss their personal experiences. The second is to broaden the selection of the participants. The third is to have at least a pair of interviewers. Having a pair of interviewers, in addition to using a recording device, would decrease the bias of the interviewer and increase feedback on the interpretation of data. These alterations to the key elements of the technique will improve the discussions and the value of the responses of the interviewees.

The second objective was to explore whether the convergent interview technique would bring out new perceptions not discovered by other qualitative collection techniques. The results of the interview revealed three new perceptions about SDS which is minimally discussed in the practice of head and neck surgery. The convergent interview revealed that: (1) SDS is not included in resident training; (2) there is a cultural resistance to the SDS technology; and (3) the field of surgical design and simulation is a work in progress.

## Abbreviations

(VSP): Virtual surgical planning; (SDS): Surgical design and simulation; (3D): Three dimensions; (MMRL): Medical modeling research laboratory; (iRSM): Institute for reconstructive sciences in medicine.

## Competing interests

The authors declare that they have no competing interests.

## Authors’ contributions

HL, JW, HS, BH and PB all conceived the study, participated in its design and coordination and drafted the manuscript. The present study was one of three studies done to complete a thesis for a Master of Science in Rehabilitation Science focusing in Surgical Design and Simulation (SDS). JW, HS, BH and PB all participated as supervisory committee members for the MSc of HL. All authors read and approved the final manuscript.

## Authors’ information

Heather Logan (HL) - MSc, BDes

HL is a Surgical Design Simulationist at the Institute for Reconstructive Sciences in Medicine (iRSM). She completed her Master of Science in Rehabilitation Science with a focus in Surgical Design and Simulation in 2011 at the University of Alberta. HL divides her time between clinical work in facial prosthetics, surgical design and simulation and research. Her research involves developing and refining surgical design methods and developing digital processes for facial prosthetic fabrication.

Dr. Johan Wolfaardt - BDS, MDent (Prosthodontics), PhD

JW is a Director of the Institute of Reconstructive Sciences in Medicine (iRSM) and is appointed as a Full Professor in the Faculty of Medicine and Dentistry, University of Alberta, Canada. His clinical and research interests are in the area of maxillofacial prosthetics with particular emphasis in the area of head and neck reconstruction, osseointegration and treatment outcomes. JW has led the development of the research program at COMPRU. His research interests involve treatment outcomes, digital technologies in head and neck reconstruction and biomechanics of osseointegrated implants. JW has published over 80 papers in refereed journals and contributed to a variety of texts. He has lectured both nationally and internationally on maxillofacial prosthetics, osseointegration in head and neck reconstruction, challenges of introduction of advanced digital technology, knowledge work, teamwork and quality management. JW is elected to the Boards of the International Society of Maxillofacial Rehabilitation, the American Academy of Maxillofacial Prosthetics and the International College of Prosthodontists.

Dr. Hadi Seikaly - MD, FRCSC

HS is a Professor of Surgery in the Department of Surgery (University of Alberta), Director of the Division of Otolaryngology Head and Neck Surgery, and the Zone Section Head for Otolaryngology Head and Neck Surgery. In addition, he is presently the president of the University Hospital Medical Staff Society, serves on several local, national, and international administrative committees and is on the Executive Council of the Canadian Society of Otolaryngology. HS graduated from the University of Toronto medical school and completed his residency training at the University of Alberta in Otolaryngology Head and Neck Surgery. He then obtained fellowship training at the University of Texas Medical Branch in advanced head & neck oncology, microvascular reconstruction and facial cosmetic surgery. Dr. Seikaly returned to the University of Alberta as an attending in the division of Otolaryngology, department of surgery in 1996 where he has been active in teaching, patient care, and research.

HS continues to have a large practice dedicated to head, neck and skull base oncology and reconstruction. His research interests include functional surgical and reconstructive outcomes, microvascular head and neck reconstruction, submandibular gland transfer and medical modeling as it applies to the head and neck region.

He has published more than 50 papers in peer reviewed journals and numerous chapters.

Dr. Pierre Boulanger – Ph.D., P.Eng

PB worked for 18 years at the National Research Council of Canada as a senior research officer where his primary research interests were in 3D computer vision, rapid product development, and virtualized reality systems. He now holds a double appointment as a professor at the University of Alberta in the Department of Computing Science and in the Department of Radiology and Diagnostic Imaging (Faculty of Medicine). PB is the Director of the Advanced Man Machine Interface Laboratory as well as the scientific director of the Alberta Radiological Visualization Center. His main research topics and teachings are on virtualized reality systems and medical imaging. He is also a Principal Investigator for Stereo IPTV at TRLabs. PB has published more than 260 scientific papers in various journals and conferences. PB is on the editorial board of two major academic journals as well as on many international committees and frequently gives lectures on rapid product development and virtualized reality. On the commercial side, PB is the President of PROTEUS Consulting Inc., an Alberta-based consulting firm specialized in Virtual Reality Applications.

Dr. Bill Hodgetts – Ph.D.

BH obtained his B.A. in Psychology and his M.Sc. in Audiology at the University of Western Ontario. He received his Ph.D. in Rehabilitation Sciences at the University of Alberta. BH is an Associate Professor in the Department of Speech Pathology and Audiology at the University of Alberta where he teaches in the areas of hearing science/audiology and research methods and statistics. He has a joint appointment with the Institute for Reconstructive Sciences in Medicine (iRSM), where he is program director of Bone Conduction Amplification. BH’s research involves developing and refining the selection, verification, and validation of fitting procedures for BAHA (bone anchored hearing aid). He also has a research interest in bone anchored hearing aids and noise exposure from MP3 Players.
